# Risk factors for suicidal attempts in a sample of outpatients with treatment-resistant depression: an observational study

**DOI:** 10.3389/fpsyt.2024.1371139

**Published:** 2024-03-22

**Authors:** Serena Chiara Civardi, Filippo Besana, Giovanni Carnevale Miacca, Filippo Mazzoni, Vincenzo Arienti, Pierluigi Politi, Natascia Brondino, Miriam Olivola

**Affiliations:** ^1^ Department of Brain and Behavioural Sciences, University of Pavia, Pavia, Italy; ^2^ Department of Mental Health and Addictions, Azienda Socio-Sanitaria Territoriale (ASST), Pavia, Pavia, Italy

**Keywords:** treatment-resistant depression, suicide risk, suicidal attempt, antidepressant therapy, clinical assessment

## Abstract

**Introduction:**

Treatment-resistant depression (TRD) is commonly defined as the failure of at least two trials with antidepressant drugs, given at the right dose and for an appropriate duration. TRD is associated with increased mortality, compared to patients with a simple major depressive episode. This increased rate was mainly attributed to death from external causes, including suicide and accidents. The aim of our study is to identify socio-demographic and psychopathological variables associated with suicidal attempts in a sample of outpatients with TRD.

**Material and methods:**

We performed a monocentric observational study with a retrospective design including a sample of 63 subjects with TRD referred to an Italian outpatient mental health centre. We collected socio-demographic and psychopathological data from interviews and clinical records.

**Results:**

77.8% of the sample (N=49) were females, the mean age was 49.2 (15.9). 33.3% (N=21) of patients had attempted suicide. 54% (N=34) of patients had a psychiatric comorbidity. Among the collected variables, substance use (p=0.031), psychiatric comorbidities (p=0.049) and high scores of HAM-D (p=0.011) were associated with the occurrence of suicide attempts. In the regression model, substance use (OR 6.779), psychiatric comorbidities (OR 3.788) and HAM-D scores (OR 1.057) were predictive of suicide attempts. When controlling for gender, only substance use (OR 6.114) and HAM-D scores (OR 1.057) maintained association with suicide attempts.

**Conclusion:**

The integrated treatment of comorbidities and substance abuse, which involves different mental health services, is fundamental in achieving the recovery of these patients. Our study supports the importance of performing a careful clinical evaluation of patients with TRD in order to identify factors associated with increased risk of suicide attempts.

## 1 Introduction

All over the world about 300 million people suffer from major depressive disorder (MDD) (1). The World Health Organization (WHO) has identified MDD as the primary cause of disability burden, leading to reduced productivity, heightened healthcare expenses, and, most significantly, hindrances in achieving a fulfilling and enriching life (2). The advent of antidepressant medications has brought about a transformative shift in the treatment of major depression. Unfortunately, however, about 60% of patients do not show an adequate response to first line pharmacological treatments and 30% respond poorly to different trials with various antidepressants (3). The extreme variability in antidepressant treatment response is likely due to neurobiological and environmental factors (4).

Treatment-resistant depression (TRD) is commonly defined by the lack of positive response to at least two types of antidepressant medication, administered at the correct dosage and for a suitable duration (5). However, experts still do not agree on the definition of appropriate dose and appropriate treatment duration (6) and a consensus definition of TRD has not yet been reached. There is also little consensus about the best tools to diagnose TRD and measure its outcomes. These limitations hampered the possibility to compare and summarize study results, thus limiting the possibility to define clinical guidelines (7).

Several studies have reported that TRD could be associated with increased mortality (8, 9), although the sample sizes were small and follow-up times have been relatively short. A Swedish population-based study considering 118,774 individuals diagnosed with depression reported an overall mortality 1.35 times higher among patients with TRD compared to individuals with MDD (10). The increased rate was mainly attributed to external causes, including suicide and accidents.

A systematic review of suicidality in TRD found an overall incidence of completed suicides of 0.47 per 100 patients/year and of attempted suicides of 4.66 per 100 patients/year (95% CI: 3.53-6.23) (11). These are respectively twice and ten times greater than those found in non-resistant patients: 0.22 completed and 0.43 attempted suicides per 100 patients/year (12). In general, several studies pointed out that 30% of patients with TRD had one or more suicide attempts (13). Another recent study (14) dealing with suicidality in the context of major depression found that individuals with TRD had higher suicide rates compared to those who were diagnosed with MDD. Previous studies also highlighted that suicide related mortality in TRD was higher than in MDD even when depressive symptoms were classified as “mild” (15, 16). Furthermore, most authors underlined that the type of suicide attempt, which can be classified in impulsive, frequent or well-planned (17), is almost never reported. This hampers the study of underlying moderators of the high suicide risk observed in TRD. For example, suicide attempts classified as impulsive may indicate a decreased impulse control in TRD patients or an increase of impulsiveness that might be responsive to a different treatment. Another possible interpretation is that TRD patients could be aware of the limited therapeutic options for future improvement, which could lead to a higher proportion of well-planned suicide attempts, compared to non-resistant patients.

Patients with TRD often have comorbid personality disorders and this comorbidity could represent the underlying moderator of frequent suicide attempts in this subset of TRD patients. In a recent review article (18) a significant impact to development of TRD is determined by the co-occurring diagnosis of a personality disorder, as personality disorders in general respond poorly to pharmacological treatment. In particular, borderline personality disorder is characterised by high levels of impulsivity, unstable self-image, feeling of emptiness and extreme mood instability. It has been reported that depressive disorder with comorbid borderline personality disorder (BPD) shows greater treatment resistance and worse functional impairment (19).

Based on these premises, our study aims to identify which socio-demographic and psychopathological data are associated with suicidal attempts in a sample of TRD outpatients.

## 2 Materials and methods

### 2.1 Characteristics of the study

We performed a monocentric observational study with a retrospective design including 63 outpatients aged between 19 and 79 years diagnosed with major depression (ICD-10 criteria). Patients were in charge of an Italian mental health outpatient centre. Subjects were labelled as “treatment resistant” based on their psychopharmacological history. We accepted the definition of TRD as the failure of at least two antidepressants prescribed at an appropriate dose and duration (5).

### 2.2 Assessment instruments

Personal and clinical data were collected during interviews. The content of each clinical interview, as part of the general clinical practice, is reported in the patient’s personal clinical record.

We considered the following clinical data: age, gender, occupational and marital status, family history of psychiatric disorders, present and past psychopharmacological therapies, substance and/or alcohol use disorder, presence of comorbid personality disorders, number of suicide attempts, duration of current and past depressive episode.

Psychopathology, with an emphasis on mood symptoms, was assessed by means of the following scales, administered at baseline, as a test battery specific for patients diagnosed with TRD: the Montgomery-Asberg Depression Rating Scale (MADRS) (20), the Hamilton Rating Scale for Anxiety (HAM-A) (21) and Depression (HAM-D) (22), the Brief Psychiatric Rating Scale (BPRS) (23), the Koukopoulos Mixed Depression Rating Scale (KMDRS) (29). The diagnosis of personality disorder was established using the Structured Clinical Interview (SCID II, 24).

#### 2.2.1 Montgomery-Asberg depression rating scale

This clinician-rated scale is designed to measure depression severity and detect changes due to antidepressant treatments. The scale consists of 10 items, scored from 0 (symptom not present or normal) to 6 (severe or continuous presence of the symptom), for a maximum total score of 60. The MADRS evaluates apparent sadness, reported sadness, inner tension, sleep, appetite, concentration, lassitude, inability to feel (interest level), pessimistic thoughts, and suicidal thoughts (25).

#### 2.2.2 Hamilton rating scale for anxiety

HAM-A is represented by 14 items. Every item individually encompasses a group of symptoms. This scale estimates both psychic anxiety and somatic anxiety including mental agitation and psychological distress; and physical complaints related to anxiety, respectively. The item score ranges from 0 to 4. The higher the score, the more severe the anxiety. The total score ranges from 0 to 56. In this scale, score < 17 indicates mild severity, 18-24 represents mild to moderate severity, and 25-30 refers to moderate to severe condition (26).

#### 2.2.3 Hamilton rating scale for depression

The HAM-D (22) is a clinical interview for the severity of depressive symptoms and one of the most frequently used outcome measures of depression in adults. We used the 17-item form (assessing depressed mood, suicide, insomnia initial - middle – delayed, work and interests, retardation, agitation, anxiety psychic – somatic, somatic gastrointestinal, somatic general – genital, hypochondriasis, insight, loss of weight). Items are scored from 0 to 4 or 0 to 2 depending on the symptom assessed, with higher scores indicating greater symptom pathology (27).

#### 2.2.4 Brief psychiatric rating scale

The Brief Psychiatric Rating Scale (BPRS) was developed to measure changes in a comprehensive set of psychopathologic symptoms present in major psychiatric diagnoses. The items assess the following symptom domains: 1. somatic concern, 2. anxiety, 3. emotional withdrawal, 4. conceptual disorganization, 5. feelings of guilt, 6. tension, 7. mannerisms and posturing, 8. grandiosity, 9. depressive mood, 10. hostility, 11. suspiciousness, 12. hallucinatory behavior, 13. motor retardation, 14. uncooperativeness, 15. unusual thought content, 16. blunted affect, 17. excitement, and 18. disorientation. Each item is rated on a seven-point Likert scale, ranging from “1” (not present) to “7” (extremely severe). Thus, the sum score ranges between 18 and 126, with a higher score indicating more severe symptomatology (28).

#### 2.2.5 Koukopoulos mixed depression rating scale

The Koukopoulos Mixed Depression Rating Scale (KMDRS) is a scale specifically created to assess mixed depression. Koukopoulos and collaborators developed and validated specific criteria such as the presence of a depressive episode plus absence of retardation, talkativeness, psychic agitation on inner tension, description of suffering from spells of weeping, racing or crowded thoughts, irritability or unproved rage, mood lability or marked reactivity, early insomnia. It includes 14 items. Items 1-4, 6, 8-11, 13-14 are evaluated according to a Likert-type scale whose scores range from 0 to 3; items 5, 7 and 12 have a score range from 0 to 6. In each item, 0 indicates the absence of the symptom relating to the single item, while 3 or 6, depending on the item, represents the maximum severity level of the relevant item. Therefore, the scale ranges from a minimum of 0 to a maximum of 51 (29).

### 2.3 Statistical analysis

First, descriptive analyses of the variables considered were carried out. Chi-square test was performed for the nominal variables. Paired sample t-test or Mann-Whitney were performed (according to the type of distribution) for the quantitative and ordinal variables, according to the presence or absence of suicidal attempts. A two-tailed p value <0.05 was regarded as statistically significant. For the variables in which statistical significance was found, we performed regression analyses with suicidal attempts as outcome variable and the retrieved variables as the independent predictors. Data were analysed using the Jamovi program (Version 2.3, 30).

## 3 Results

Clinical characteristics of the sample are presented in **Table 1**. 77.8% of the sample (N=49) were females, and the mean age was 49.2 years (SD 15.9). 47.6% of the sample (N=30) were in treatment with Esketamine. In our sample, 21 (33.3%) patients attempted suicide. 32 patients (50.8%) had a psychiatric comorbidity. Of these, 23 patients (36.5% of the sample) had a personality disorder. Detailed treatment regimens of each patient included in the study are described in **Supplementary Table 1**.

**Table 1:** General characteristics of the sample.

Socio-demographic and psychopathological variables features of the sample (N=63)	
**Gender**	49 F (77.8%), 14 M (22.2%)
**Age (Mean, SD, range)**	49.2 (15.9), 19-79
**Marital status**	single 31 (49.2%) married/in a relationship 32 (50.8%)
**Children**	Yes 34 (56%) No 29 (46%)
**Education**	Primary school 4 (6.3%) Secondary school 20 (31.7%) High school 31 (49.2%), University degree 8 (12.7%)
**Employment status**	Unemployed 22 (34.9%) Employed 26 (41.3%) Student 5 (7.9%) Retired 10 (15.9%)
**Familiar history for mental disorders**	Yes 32 (50.8%) No 31 (49.2%)
**Age of first depressive episode (Mean, SD, range)**	34.2 (17.2) 11-79
**Psychiatric Comorbidities**	Yes 34 (54%) No 29 (46%)
**Psychiatric Comorbidities (ICD-10 or SCID-II criteria)**	None 29 (46%) Personality disorder 23 (36.5%): borderline 10, narcissistic 2, histrionic 1, dependent 2, obsessive-compulsive 1, NOS 7 Eating disorder 3 (4.8%) Bipolar disorder 3 (4.8%) PTSD 1(1.6%) Autism spectrum disorder 1 (1.6%) Generalised anxiety disorder 1 (1.6%) Cyclothymic disorder (1.6%) OCD 1 (1.6%)
**Substance use**	Yes 27 (42.9%) No 36 (57.1%)
**Suicide attempts**	Yes 21 (33.3%) No 42 (66.7%)
**Main antidepressant prescribed**	Sertraline 7 (11.1%) Venlafaxine 6 (9.5%) Vortioxetine 6 (9.5%) Trazodone 3 (4.8%) Duloxetine 2 (3.2%) Fluoxetine 2 (3.2%) Escitalopram 2 (3.2%) Paroxetine 2 (3.2%) Mirtazapine 1 (1.6%) Citalopram 1 (1.6%) Clomipramine 1 (1.6%) Esketamine 30 (47.6%)
**Concomitant therapies: antipsychotics**	Yes 44 (69.8%) No 19 (27.3%)
**Concomitant therapies: mood stabilisers**	Yes 35 (55.6%) No 28 (44.4%)
**MADRS (baseline)**	27.0 (13.7)
**HAM-A (baseline)**	25.2 (15.9)
**HAM-D (baseline)**	22.6 (17.6)
**BPRS (baseline)**	47.3 (16.7)
**KMDRS (baseline)**	13.6 (6.75)

Univariate analyses are described in **Table 2**. Substance use, personality disorder, psychiatric comorbidity and higher HAM-D scores were significantly more frequent among suicide attempters. All other variables were not significantly different between the two groups.

**Table 2:** Univariate analysis.

Variable		Suicidal Attempt (No) N=42	Suicidal Attempt (Yes) N=21	P	Chi-square/U di Mann-Whitney
**Gender**	F	31	18	0.284	1.15*
	M	11	3		
**Marital status**	Single	21	10	0.86	0.031*
	Married/in a relationship	21	11		
**Children**	Yes	22	12	0.721	0.128*
	No	20	9		
**Age (mean, SD, range)**		51.6 (15.3) range 22-79	44.2 (16.1) range 16-72	0.092	325^
**Education**	Primary School	4	0	0.059	7.43*
	Secondary school	12	8		
	High school	18	13		
	Degree	8	0		
**Occupation**	Unemployed	15	7	0.319	3.52*
	Employed	15	11		
	Retired	9	1		
	Student	3	2		
**Substance use**	Yes	14	13	**0.031**	4.67*
	No	28	8		
**Psychiatric comorbidity**	Yes	19	15	**0.049**	3.87*
	No	23	6		
**Personality disorder**	Yes	11	12	**0.016**	5.79*
	No	31	9		
**Personality disorder (type)**	Borderline	4	6	0.563	4.72*
	Narcissistic	1	1		
	Histrionic	1	0		
	Dependent	1	1		
	Obsessive-compulsive	1	0		
	NOS	3	4		
**Previous major depressive episodes (N, SD)**		4.22 (4.72)	4.05 (4.40)	0.897	0.130^
**Family history for mental disorders**	Yes	21	11	0.931	0.008*
	No	20	10		
**Concomitant therapies: antipsychotics**	Yes	29	15	0.846	0.038*
	No	13	6		
**Concomitant therapies: mood stabilisers**	Yes	22	13	0.473	0.514*
	No	20	8		
**HAM-A**		23.0 (14.6)	29.8 (17.9)	0.111	-1.162^
**HAM-D**		18.7 (13.4)	30.4 (22.4)	**0.012**	**-2.59^**
**MADRS**		25.7 (12.4)	29.6 (15.9)	0.289	-1.07^
**BPRS**		46.4 (16.8)	48.9 (17.3)	0.579	-0.558^
**KMDRS**		14.4 (6.67)	12.3 (6.86)	0.249	1.16^

*chi-square test; ^t- test or Mann-Whitney test. Bold characters indicate statistically significant values (p<0.05).

We then constructed a regression model using suicidal attempts as the dependent variable and all other variables as independent predictors (**Table 3**). The resulting model explained 34.8% of the variance (R^2^N=0.348, AIC=70.0, p<0.001). The independent predictors were the presence of a psychiatric comorbidity, substance use and HAM-D scores (**Table 3**). We also performed a regression model with the same variables and gender as a controlling factor (**Table 4**). In this model, the explained variance was also slightly increased and substance use and HAM-D were the only predictive variables for suicidal attempts (R^2^N=0.354, AIC 71.7 p<0.001).

**Table 3:** Predictors of suicidal attempts-model 1.

Predictor	SE	Z	P	OR	95% CI	
**Psychiatric comorbidity**	1.332	**2**	**0.046**	3.788	1.026	13.994
**Substance use**	0.021	**2.72**	**0.007**	6.779	1.705	13.994
**HAM-D**	0.0212	**2.62**	**0.009**	1.057	1.014	1.102

chi-square test; ^t- test or Mann-Whitney test. Bold characters indicate statistically significant values (p<0.05).

**Table 4:** Predictors of suicidal attempts-model 2.

Predictor	SE	Z	P	OR	95% CI	
**Psychiatric comorbidity**	0.678	1.847	0.065	3.495	0.926	13.188
**Substance use**	0.720	**2.514**	**0.012**	6.114	1.49	25.088
**HAM-D**	0.0213	**2.631**	**0.009**	1.057	1.014	1.103
**Gender**	0.933	-0.571	0.568	0.587	0.094	3.654

Bold characters indicate statistically significant values (p<0.05).

## 4 Discussion

Our research focused on identifying the risk factors associated with suicide attempts among individuals diagnosed with Treatment-Resistant Depression (TRD) who were receiving care at an outpatient mental health facility in Italy. We observed that substance use was associated with a higher rate of suicide attempts. Additionally, psychiatric comorbidities, namely borderline personality disorder, were also associated with a higher rate of suicide attempts.

Notably, about 30% of individuals with TRD make suicide attempts at least once in their lifetime, as reported by several studies, twice as much as in non-resistant depression (31, 32). This datum is in line with the percentage registered in our sample (33%, n=21). This underscores the need of vigilant clinical monitoring of TRD patients, considering that a close psychiatric follow-up following a suicide attempt has demonstrated an antisuicidal protective effect (33).

In our sample, 77.8% were female patients. This prevalence is higher than in previous studies, but in line with generally higher prevalence of TRD in females (34), as well as in MDD in general (35, 36). For example, Herlein and colleagues identified a 62.3% prevalence of females in their sample, while another study recorded a female prevalence rate of 52.6% (37). In our sample, patients who have attempted suicide are on average younger (44.2 vs 51.6 years old), as previous research has already outlined (38).

The association between substance use and suicidal attempts that we observed is in line with previous research: one nested case-control study (39) based on a Swedish nation-wide register of TRD patients observed a correlation of substance use disorders, personality disorders, and anxiety disorders with attempted suicides. Our hypothesis is that substance use disorder may be a proxy of impulsiveness which could result in suicide attempts. Our findings emphasise the importance of implementing primary and secondary prevention strategies regarding psychoactive substance use, as well as to enhance cooperation between general psychiatry and addiction mental health services (40).

In our study, psychiatric comorbidities have been associated with suicide attempts. In a recent study, patients with TRD, compared to patients with MDD have a higher rate of psychiatric comorbidities, a longer duration of depressive episodes and three times the number of inpatient bed-days (41). These findings stress the importance of early identification of patients with MDD and high risk of TRD, in order to target health care efforts (42). Taking into account the HAM-D rating scale, we found that higher scores (i.e. worse depressive symptomatology) were associated with suicide attempts. This datum has been already highlighted by previous studies (43, 44). In the same way, it has been demonstrated that patients with TRD have on average higher HAM-D scores than patients with major depression, as well as a longer duration of illness (45).

Furthermore, we found an association between personality disorders and suicidal attempts. This finding has been already outlined by Reutfors et al. (39), who found as independent risk factors for suicidal attempts a history of suicide attempts, substance abuse, personality disorders, and somatic comorbidity. This finding implies a careful assessment of personality disorders through evidence-based instruments, as well as an adequate treatment with a targeted psychotherapy (46, 47). As far as our sample is concerned, the most represented among personality disorders was borderline personality disorder (BPD, n=10, 15.9% of the total sample and 43.5% of personality disorders, respectively). Depressive disorder and borderline personality disorder are often comorbid. The high impulsivity and the poor mentalizing skills that are core feature of BPD often lead to self-injurious behaviours and suicide attempts. As well as TRD, borderline personality disorder responds poorly to conventional pharmacological treatments. However, we didn’t find a specifical association between BPD and suicidal attempts, since patients with BPD were nearly equally distributed in the two categories. This may also be related to the relatively small sample size of our study, and study with a bigger sample could better define this association.

The topic of suicidality among patients with a primary diagnosis of treatment-resistant depression necessitates meticulous investigation, given the heightened prevalence of suicidal thoughts, attempts, and completed suicides in individuals with TRD as opposed to those with Major Depressive Disorder (MDD) (48). TRD represents a clinical challenge in psychiatry, since it is associated with a loss of quality of life, a lower productivity, more hospitalizations and higher healthcare costs (49, 50).

Some pharmacological therapies have shown promising results in treating TRD. For example, esketamine, the levo enantiomer of ketamine, has been approved for the treatment of TRD and can be administered as a nasal spray. This drug has proven generally safe and well tolerated and has provided meaningful and rapid impact on reducing depressive symptoms and suicide ideation (51). A possible beneficial effect on suicidal behaviour in TRD patients has also been suggested for lithium. In a meta-analysis by Cipriani and colleagues (52) authors found that lithium helped reducing suicide risk in patients with mood disorders. It was hypothesised that it may exert its antisuicidal effects by reducing relapse of mood disorder and also by decreasing aggression and possibly impulsivity, which might be another mechanism mediating the antisuicidal effect. Regarding TRD, evidence about lithium’s antisuicidal effects is still poor.

As far as non-pharmacological treatments are concerned, various studies have focused on Repetitive Transcranial Magnetic Stimulation (rTMS) that is a non-invasive brain stimulation technique used to treat mood disorders, including TRD, but also other mental illnesses such as obsessive-compulsive disorder (OCD) and borderline personality disorder (BPD). A recent meta-analysis by Chen et al. (53) found that rTMS significantly reduced suicidal ideation and improved depressive symptoms. Focusing on suicidal ideation, this was reduced after rTMS in patients with major depressive disorder but not in those with TRD.

Our study has several limitations. First of all, the sample size was quite small, thus limiting the generalizability of the results. In this regard, future studies with larger sample sizes and a prospective design could provide a more precise insight into our findings. Moreover, our study did not collect other relevant parameters related to suicidal attempts, such as the number of hospitalizations and the modality of attempted suicide, which is known to be a relevant diagnostic and prognostic factor (54). Secondly, we did not use a specific appropriate assessment instrument for suicidal ideation (48, 55). These limitations are mainly due to the research design which was carried out at a public mental healthcare facility, in a “real world” setting. Therefore, assessments were not specifically designed to investigate specific psychopathological domains. A larger sample size and a more careful assessment of these features could lead to more accurate results. Lastly, we did not perform a psychopathological assessment at follow-up with the rating scales performed at baseline. Such finding would have allowed us to highlight symptomatic changes at follow-up and any correlations with suicidal behaviors. In future research, it could be interesting, as we found an association between HAM-D scores and suicide attempts, to analyze separately every single item of this scale with respect to suicidality in a more dimensional approach. Indeed, the National Institute of Mental Health Collaborative Depression study highlighted three group of symptoms (1, anhedonia, hopelessness; 2, anxiety, agitation, panic; 3, aggression, impulsivity) as more predictive of suicide than either diagnoses or syndrome (56).

Our study supports the importance of performing a careful clinical evaluation of patients with treatment-resistant depression and of raising awareness among clinicians to prevent pseudo-resistance and to identify factors associated with increased severity of symptoms. In this regard, it would be useful to insert standardised evaluation such as the HAM-D scale (which is overall not time-consuming) in clinical practice. Additionally, assessment of personality disorders as well as potential dysfunctional personality traits deserve a place in clinical practice (57). On the other hand, clinicians should strictly adhere to pharmacological guidelines, following the correct doses of each medication and the adequate duration of the treatment in order to identify TRD correctly and perform a differential diagnosis between TRD and refractory depression, that is a form of depressive disorder that has not shown adequate response to any treatment (58). For a more personalised and targeted therapy, pharmacogenomic tests hold great promise for future routine clinical practice but now they are mostly limited to specialised services (59). In conclusion, clinical practice should incorporate personalized medicine principles in order to choose more effective pharmacological and psychosocial therapeutic strategies.

## Data availability statement

The data analyzed in this study is subject to the following licenses/restrictions: The dataset includes privacy-related information. Requests to access these datasets should be directed to Filippo Besana; filippo.besana01@universitadipavia.it.

## Ethics statement

Ethical approval was not required for the study involving humans in accordance with the local legislation and institutional requirements. Written informed consent to participate in this study was not required from the participants or the participants’ legal guardians/next of kin in accordance with the national legislation and the institutional requirements.

## Author contributions

SC: Formal analysis, Writing – original draft, Writing – review & editing, Data curation, Software. FB: Formal analysis, Writing – original draft, Writing – review & editing, Conceptualization. GM: Data curation, Writing – original draft, Writing – review & editing. FM: Conceptualization, Data curation, Writing – original draft, Writing – review & editing. VA: Data curation, Writing – original draft, Writing – review & editing. PP: Supervision, Visualization, Writing – original draft, Writing – review & editing. NB: Formal analysis, Supervision, Writing – original draft, Writing – review & editing. MO: Conceptualization, Methodology, Supervision, Writing – original draft, Writing – review & editing.
