# Supplementary material for: Risk factors for suicidal attempts in a sample of outpatients with treatment-resistant depression: an observational study
Source: Front Psychiatry. 2024 Mar 22;15:1371139. doi: 10.3389/fpsyt.2024.1371139 (PMC10995380; doi:10.3389/fpsyt.2024.1371139)
Supplement: Supplementary file 1 [file Table_1.docx]

**Supplementary material-Pharmacotherapy of the patients included in the study (N=63)**

| 7 (11.1%) Antidepressant (SSRI)+Atypical antipsychotic |
| --- |
| 6 (9.5%) Antidepressant (SSRI)+Dopamine partial agonist |
| 1 (1.6%) Antidepressant (SARI) |
| 6 (9.5%) Antidepressant (SNRI)+Dopamine partial agonist |
| 4 (6.3%) Antidepressant (SNRI)+Atypical antipsychotic |
| 3 (4.8%)Antidepressant (SNRI)+ Antidepressant (SARI) |
| 6 (9.5%) Antidepressant (SARI)+Dopamine partial agonist |
| 1 (1.6%) Antidepressant (Vortioxetine) |
| 2 (3.2%) Antidepressant (Vortioxetine)+ Dopamine partial agonist |
| 7 (11.1%) Esketamine+Antidepressant (SSRI) |
| 6 (9.5%) Esketamine+Antidepressant (SSRI)+Dopamine partial agonist |
| 5 (7.9%) Esketamine+Antidepressant (SNRI) |
| 2 (3.2%) Esketamine+Antidepressant (SNRI)+Dopamine partial agonist |
| 3 (4.8%) Esketamine+ Antidepressant (Vortioxetine) |
| 3 (4.8%) Esketamine+Antidepressant (SNRI)+Mood stabiliser (Valproic acid)+Dopamine partial agonist |
| 1 (1.6%) Esketamine+Antidepressant (SSRI)+Mood stabiliser (Carbolithium)+Atypical antipsychotic |
